# Prognostic Value of the Global Left Ventricular Contractility Index in Patients with Severe Mitral Regurgitation and Preserved Left Ventricular Ejection Fraction

**DOI:** 10.3390/jcdd12060227

**Published:** 2025-06-13

**Authors:** Tony Li, Vinay B. Panday, Jessele Lai, Nicholas Gao, Beth Lim, Aloysius Leow, Sarah Tan, Quek Swee Chye, Ching Hui Sia, William Kong, Tiong Cheng Yeo, Ru San Tan, Liang Zhong, Kian Keong Poh

**Affiliations:** 1 Department of Cardiology, National University Heart Centre Singapore, 1E Kent Ridge Road, Tower Block Level 9, Singapore 119228, Singapore; 2 Yong Loo Lin School of Medicine, National University of Singapore, 10 Medical Dr, Singapore 117597, Singapore; 3 Division of Paediatric Cardiology, Department of Paediatrics, Khoo Teck Puat-National University Children’s Medical Institute, National University Hospital, Singapore 119228, Singapore; 4 National Heart Center Singapore, 5 Hospital Dr, Singapore 169609, Singapore

**Keywords:** mitral regurgitation, global left ventricular contractility index, heart failure, preserved ejection fraction

## Abstract

Introduction: Assessment of left ventricular (LV) systolic function is important in valvular heart disease. The global LV contractility index, dσ*/dt_max_, is load-independent and has been reported to be associated with clinical outcomes in heart failure and aortic stenosis. We aim to assess if dσ*/dt_max_ could predict adverse outcomes in patients with severe mitral regurgitation (MR). Methodology: We studied dσ*/dt_max_ in a cohort of 127 patients with isolated severe primary MR and preserved LVEF ≥ 60%. Patients with prior valvular intervention or concurrent valvular disease were excluded. We tested dσ*/dt_max_ against a composite of adverse outcomes including all-cause mortality, heart failure hospitalization, and mitral valve intervention. Results: The cohort had a mean age of 58 years old and was predominantly male. Of the 127 patients, eight (6.3%) needed subsequent hospitalization for heart failure, while 30 (23.6%) and 11 (8.7%) patients underwent mitral valve repair and replacement, respectively, And 14 (11.0%) passed away. Of the patients (*n* = 54 (42.5%)) who had an adverse outcome during follow-up, dσ*/dt_max_ demonstrated an independent association with composite adverse outcome, including its individual components. On ROC analysis, a cut-off of 2.15 s^−1^ was identified. Based on this cut-off, dσ*/dt_max_ retained an independent association with composite adverse outcome after adjusting for covariates including age, sex, ischemic heart disease, pulmonary artery systolic pressure, and left ventricular end systolic diameter. Conclusions: In patients with severe primary MR and preserved LVEF, reduced dσ*/dt_max_ was an independent predictor of adverse outcomes. It can be a useful addition to the armamentarium for assessing patients with severe MR.

## 1. Introduction

Mitral regurgitation (MR) is one of the most common native valvular heart diseases, and is expected to rise in prevalence with the aging population [1]. Mitral valve intervention is the only reliable therapy for severe primary mitral regurgitation. International consensus guidelines recommend such intervention in symptomatic patients with severe MR or asymptomatic patients with left ventricular systolic dysfunction as evidenced by a reduced left ventricular ejection fraction (LVEF) < 60% or increased left ventricular end systolic diameter (LVESD) > 40 mm [2,3,4]. However, these assessments are affected by the alteration of the left ventricular (LV) loading conditions and may not accurately reflect the intrinsic contractile state of the LV [5,6]. In fact, the increased preload and transmitral flow contributes to the reasons for a higher adopted cut-off of LVEF 60% instead of 50% as in assessment for potential valvular intervention in severe MR.

The global left ventricular contractility index (dσ*/dt_max_) is a novel echocardiographic index that presents an integrated assessment of LV contractile function [7,8]. This index measures the maximal rate of change in pressure-normalized LV wall stress and is primarily dependent on the outflow rate and wall volume of the LV chamber. It has been shown in previous studies to be able to predict adverse clinical outcomes in patients with heart failure with preserved ejection fraction (HFpEF) as well as aortic stenosis with preserved LVEF [9,10,11]. It is unknown if dσ*/dt_max_ is similarly able to predict adverse clinical outcomes in patients with severe MR. We aim to describe the trend of dσ*/dt_max_ in severe primary MR with preserved LVEF, and explore associations with clinical outcomes including all-cause mortality, heart failure hospitalization, and valvular intervention.

## 2. Methods

This study examines a cohort of consecutive patients with isolated severe primary MR and preserved LVEF ≥ 60% diagnosed on index echocardiography from 1 January 2014 to 31 December 2021. We included all patients with a diagnosis of severe primary MR based on the index echocardiogram. We excluded patients with other significant valvular lesions (of at least moderate severity), history of prior valvular interventions, or secondary MR based on adjudication by the reporting cardiologist. The study was approved by the National Healthcare Group Institutional Review Board (NHG DSRB 2021/00603). Echocardiographic data and relevant clinical information were obtained from the electronic medical records and databases. Follow-up data was collected up till 31 January 2022 with the average follow-up of the patients being 5.1 ± 1.8 years.

Baseline demographics and echocardiographic parameters were documented. All echocardiographic studies were performed using commercially available echocardiography systems, and images were retrospectively analyzed by experienced cardiologists according to current guidelines [12]. Retrospective review of electronic medical records was performed for identification of clinically relevant endpoints including heart failure hospitalization and all-cause mortality. We also assessed for a composite adverse outcome comprising all-cause mortality, heart failure hospitalization, and valvular intervention.

### 2.1. Assessment of dσ*/dt_max_

The global left ventricular (LV) contractility index, dσ/dt_max_*, was calculated for all patients using non-invasive echocardiographic data. This index was originally developed from fundamental mechanical engineering principles and geometric assumptions of LV structure. Since LV wall stress arises directly from sarcomeric contraction—which ultimately leads to the development of intracavitary LV pressure—the index is based on the premise that the heart’s ability to generate internal myocardial stress to eject blood reflects its contractile performance [13,14]. During systole, sarcomere activation produces myocardial wall stress, which in turn contributes to the generation of LV intracavitary pressure, commonly measured by the maximum rate of pressure rise (*dP/dt*). Importantly, wall stress begins to increase before a measurable rise in cavity pressure occurs, suggesting its potential utility as an early and sensitive marker of ventricular performance.

Using a biomechanical model, LV wall stress (*σ*) can be expressed by the following relation:

$$\sigma =P\left(\frac{3}{2V_{m}}+\frac{1}{2}\right)$$

where *σ* is wall stress, *P* and *V* are LV intracavitary pressure and volume, *Vm* is myocardial volume. Rewriting this in terms of pressure-normalized wall stress (σ*), we get

$$\frac{d\sigma^{*}}{dt_{max}}=\frac{3}{2V_{m}}{\left(\frac{dV}{dt}\right)}_{max}\;\mathrm{where}\;{\left(\frac{dV}{dt}\right)}_{max}=V_{peak}\times \frac{\pi D^{2}}{4}$$

**Figure 1:**
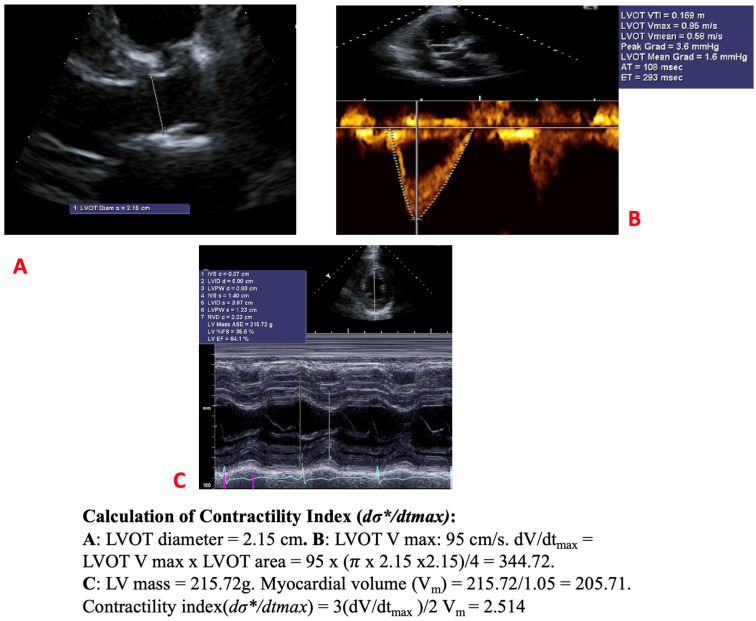
Measuring the contractility index. (**A**): LVOT diameter measurement; (**B**) Calculation of *dV/dt_max_*; (**C**) Measurement of myocardial volume *V_m_*.

Here, *dV/dt_max_* represents the maximum flow rate during systole, which is obtained through standard echocardiographic measurements. It is calculated using the peak velocity (*V_peak_*) at the left ventricular outflow tract (LVOT) from pulsed-wave Doppler and the cross-sectional area of the LVOT and D is the LVOT diameter measured in the parasternal long-axis view on echo. Myocardial volume (*V_m_*) is derived by dividing the LV mass, which is assessed via standard M-mode echocardiography, by the myocardial tissue density, assumed to be 1.05 g/mL [7,8]. A sample derivation of the global LV contractility index can be seen in Figure 1.

### 2.2. Statistical Analysis

Categorical data were expressed as frequency and percentages. Continuous variables were summarized as mean (±standard deviation). Chi-squared tests and Student’s *t*-test were used to compare variables between groups. Receiver operating characteristic (ROC) curves were used to determine the predictive power of dσ*/dt_max_ with respect to composite adverse outcome.

Univariable analysis of clinical and echocardiographic parameters was performed to identify predictors of composite adverse outcomes. Based on these findings, further multivariable Cox proportion hazards regression modeling accounting for these variables [age, sex, IHD, PASP, LVESD] was then performed to assess for the risk-adjusted association between dσ*/dt_max_ and composite clinical outcomes. While LVMI was also found to be an independent predictor of adverse composite outcomes on univariable analysis, it was not included in the multivariable analysis as myocardial volume is part of the formula to assess contractility and there might be collinearity on analysis.

Clinical outcomes were also compared by the construction of Kaplan–Meier curves. A two-tailed value of *p* ≤ 0.05 was used to reject the null hypothesis. Statistical analysis was performed using SPSS 26 (SPSS Inc., Chicago, IL, USA) and MedCalc Statistical Software version 19.2.6 (MedCalc Software bv, Ostend, Belgium; https://www.medcalc.org, accessed on 19 Febuary 2025).

## 3. Results

### 3.1. Cohort Characteristics

The clinical and echocardiographic characteristics, comorbidities, and medications of the cohort are shown in Table 1 and Table 2. A total of 127 patients with isolated severe MR were included in the study. The cohort was predominantly male (73.2%) with a mean age of 58.5 ± 13.3 years old and was followed up for a mean 5.1 ± 1.8 years. Eight patients (6.3%) needed subsequent hospitalization for heart failure, while 30 (23.6%) and 11 (8.7%) patients underwent mitral valve repair and replacement, respectively. There were 14 (11.0%) patients who passed away during the follow-up duration of the study. A composite adverse outcome comprising all-cause mortality, heart failure hospitalization, and mitral valve intervention occurred in 54 (42.5%) patients.

The composite adverse outcome was not affected by concurrent comorbidities including hypertension, hyperlipidaemia, diabetes, or ischemic heart disease. The use of diuretics, either loop diuretics or spironolactone, was associated with increased mortality and a trend towards composite adverse outcomes. This could be because patients on these medications would be expected to be more symptomatic and prone to heart failure.

Table 3 depicts the baseline echocardiographic parameters of the study cohort. As the cohort was already stratified to only include patients with LVEF ≥ 60%, LVEF was not significantly associated with the composite adverse outcomes. Various measures of LV dimensions including LVEDD, LVESD, LVEDV, LVESV, and LVMI all demonstrated correlation with adverse composite outcome. Neither the cardiac output nor PASP were associated with the composite adverse outcome. Tissue Doppler imaging (TDI)-based assessment of systolic velocity (S’) was also not associated with the composite adverse outcome.

### 3.2. Global LV Contractility Index

**Figure 2:**
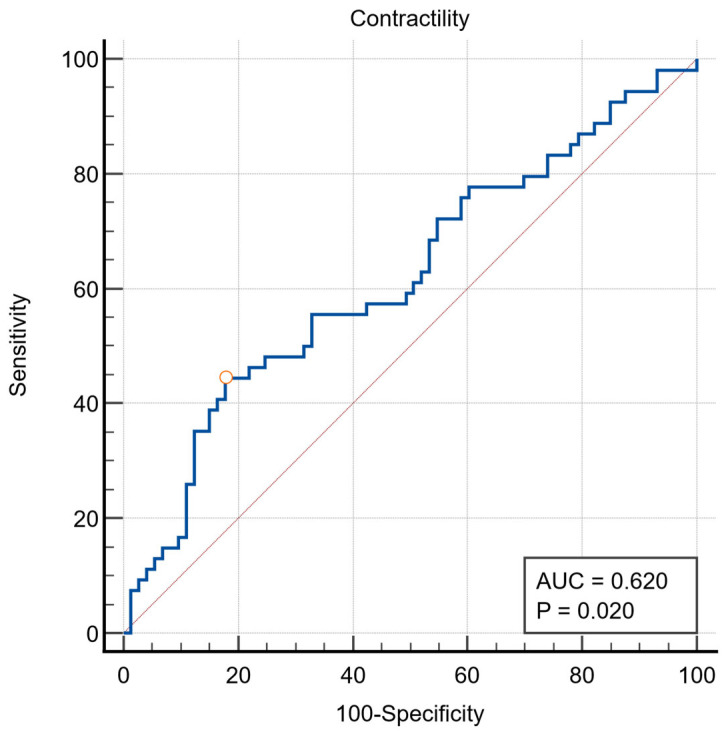
Receiver operating characteristic curve for global LV contractility index for composite adverse outcome.

The global LV contractility index was calculated based on the described methodology. It was significantly lower in patients who either suffered a composite adverse outcome or all-cause mortality alone. On receiver operating characteristic (ROC) analysis, an optimized cut-off of <2.14 s^−1^ for dσ*/dt_max_ was identified to be associated with occurrence of composite outcome (AUC 0.620, *p =* 0.020) (Figure 2). For ease of use, we rounded this off to a cut-off of <2.15 s^−1^ to represent abnormal dσ*/dt_max_ on further analysis.

**Figure 3:**
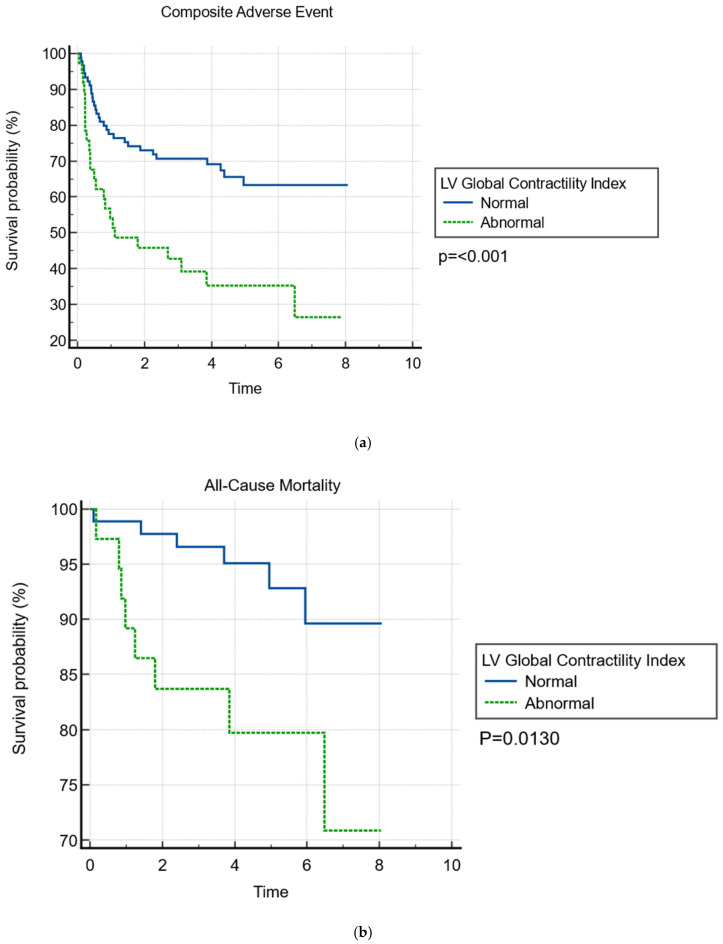

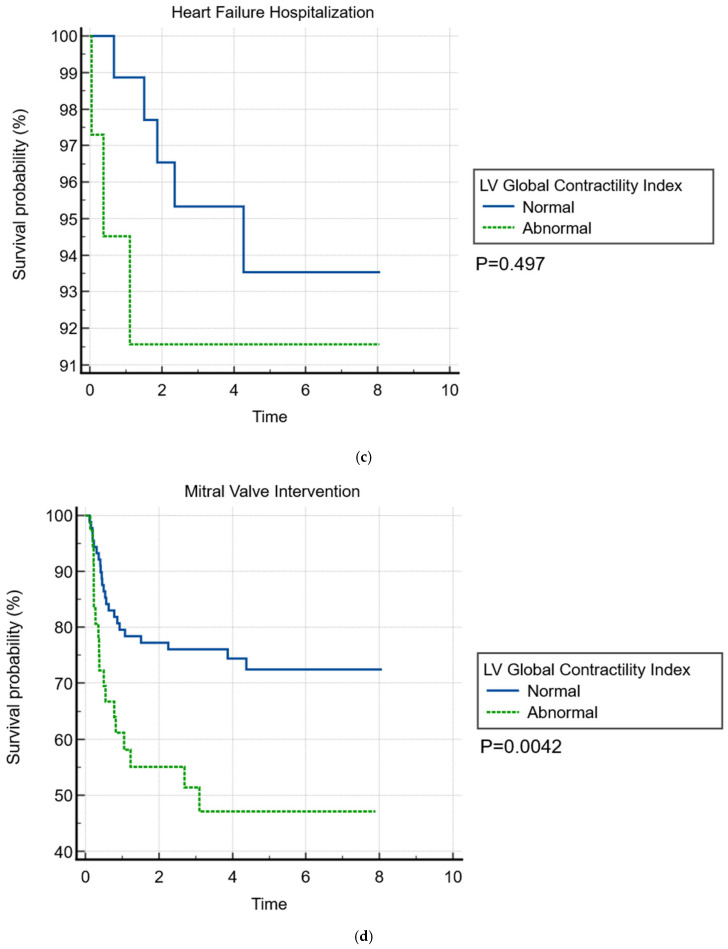
Kaplan–Meier analysis for global LV contractility index. (**a**) Against Composite Adverse Outcome, (**b**) against All-Cause Mortality, (**c**) against Heart Failure Hospitalization, (**d**) against Mitral Valve Intervention.

On Kaplan–Meier survival time-to-event analysis, patients with an abnormal global LV contractility index had a significantly higher occurrence of composite adverse events (Figure 3). On multivariable Cox regression, an abnormal global LV contractility index remained independently associated with the occurrence of composite outcomes (adjusted HR 2.056, 95% CI: 1.103–3.835, *p* = 0.023) after adjusting for covariates including age, sex, IHD, PASP, and LVESD (Table 4).

## 4. Discussion

The global LV contractility index has been studied as a novel echocardiographic parameter that can predict adverse outcomes in cardiomyopathies and valvular heart disease. To our knowledge, this is the first study to evaluate the global LV contractility index in mitral regurgitation. We further stratified the population to only include patients with severe primary MR and preserved LVEF and found that the global LV contractility index was associated with composite adverse outcomes in this population.

The goal in the assessment and management of severe primary MR is to identify appropriate patients at risk of deterioration before the point of decompensation [15]. Current guidelines recommend intervention for patients who are symptomatic or asymptomatic patients with LVEF ≤ 60% and/or LV end systolic diameter (LVESD) ≥ 40 mm which portend worse outcomes [16,17]. However, there are various limitations in using LVEF and LVESD as adverse prognostic markers.

LVEF may not be a sensitive marker of global LV systolic function in severe MR [18,19]. Firstly, LV hypertrophy and remodeling over time as an adaptive response can preserve LV contractility in the early stages. Secondly, backward ejection into the left atrium physiologically renders LVEF hyperdynamic in MR and confounds the assessment of true LV contractility. Eventually, LV remodeling may finally lead to sub-endocardial ischemia and myocardial fibrosis which causes noticeable decline in LVEF as these compensatory mechanisms are overwhelmed. Recognizing these limitations, guidelines recommend a higher cut-off of ≥60% for severe MR, in contrast to the conventional cut-off of ≥50% to identify LV systolic dysfunction as in other valvular heart diseases such as aortic stenosis. Also, a drop in LVEF is a late consequence in chronic MR and there is increasing evidence of disappointing postoperative outcomes in advanced cases of severe MR [20,21]. It would be ideal to identify patients with subtle impairment in LV performance earlier in the disease course.

The other key parameter to consider intervention in asymptomatic severe primary MR is dilated left ventricle of LVESD ≥ 40 mm. However, this has been suggested to be a weaker predictor of postoperative heart failure and survival as compared to LVEF [22,23]. This could be due to the lack of an optimal method for anthropometric normalization of LVESD and thus the parameter is not normalized to patient size [24]. The remodeling and dilatation of the LV with volume overload can also lead to distortion of the usual geometry to become more spherical, which is not captured by LVESD as it is a one-dimensional assessment [25]. Furthermore, a given LVESD could also represent either depressed or normal LV function depending on the diastolic dimensions of the case. As such, LVESD alone may not fully account for the physiologic changes that result from MR. Our study similarly supports these findings and LVESD was not significantly associated with the composite adverse outcome after adjusting for covariates.

dσ*/dt_max_ essentially measures the maximum rate of development of LV wall stress in relation to LV pressure. LV wall stress is generated intrinsically by myocardial contraction and hence, the index is directly related to the intrinsic contractility of the myocardium.

The dσ*/dt_max_ overcomes the limitations of using LVEF and LVESD by being a more sensitive marker of early cardiac dysfunction. In experimental studies, dσ*/dt_max_ was found to be preload- and afterload-independent (within physiological limits). LVEF, however, is load-dependent and hence may not be a sensitive marker of global contractility unlike the contractility index. Also, as elaborated above, LVESD alone may not capture the maladaptive response of the LV to volume overload from the MR and does not represent the contractile state of the myocardium accurately.

dσ*/dt_max_ has been studied in other disease states such as aortic stenosis and HFpEF and has shown to be a sensitive marker of early cardiac dysfunction in those disease states as well.

Recognizing that LVEF may not be the most sensitive or specific way to assess for LV contractility, there has been a variety of alternatives proposed over the years [26,27,28]. Tissue Doppler imaging (TDI) can allow for assessment of systolic velocity s’, which can reflect systolic deformation. This has been suggested to be associated with poorer outcomes in patients with severe organic MR [29]. However, TDI is largely angle-dependent, and it only measures a single component of the regional velocity vector along the scan line and may not represent global contractility. Furthermore S’ was not associated with the composite adverse outcome in our cohort.

There has also been growing interest looking into myocardial deformation parameters including strain and strain rate measurements. Specifically, LVGLS has been a parameter that has been increasingly recognized as being more sensitive for LV systolic dysfunction than LVEF, including in MR [30]. However, LV strain is not routinely performed at our center and was not available for this cohort. This does reflect the real-world challenge with LVGLS, which requires specialized software and acquisition protocols. This may limit the use of LVGLS globally, especially in under-developed ‘low- and middle-income’ countries. In our study, dσ*/dt_max_ can be easily derived from conventional echocardiographic parameters (LVOT diameter, LVOT peak velocity, and myocardial volume) without the need for specialized acquisition or software and thus can be more easily applied.

This was the first study to investigate global LV contractility index, dσ*/dt_max_, in severe MR. However, it has the inherent limitations of an observational study and can only identify correlation not causation. For this study, we also limited the analysis to patients with severe isolated MR with preserved LVEF. This therefore restricted the size of the cohort and this may limit statistical power and predictability. It would be interesting to extend the study to look into patients with moderate or perhaps even mild MR to see if an abnormal dσ*/dt_max_ is associated with adverse outcomes in these patients and identify patients that may benefit from closer monitoring. Another limitation is that we did not have access to left atrial parameters including volume and strain in this cohort.

## 5. Conclusions

In severe primary MR with preserved LVEF, a reduced dσ*/dt_max_ was independently associated with adverse composite outcomes including all-cause mortality, heart failure admission, and valvular heart intervention. The global LV contractility index can be a useful addition to the armamentarium for assessing patients with severe MR and preserved LVEF.

## Figures and Tables

**Table 1:** Baseline demographics of study population stratified by all-cause mortality.

Variables	Overall (*n* = 127)	Death *(n* = 14)	Alive (*n* = 113)	*p*-Value
Baseline Demographics				
Age (years)	58.46 ± 12.07	70.07 ± 11.19	57.03 ± 11.42	0.077
Male, *n* (%)	93 (73.2%)	11 (78.6%)	82 (72.6%)	0.758
Height	164.67 ± 8.23	162.86 ± 8.48	164.89 ± 8.21	0.742
Weight	64.83 ± 12.37	59.40 ± 13.58	65.50 ± 12.11	0.320
Smoking	29 (22.8%)	3 (21.4%)	26 (23.0%)	<0.999
Systolic Blood Pressure (mmHg)	133.15 ± 18.30	135.29 ± 26.27	132.88 ± 17.21	**0.007**
Diastolic Blood Pressure (mmHg)	74.03 ± 10.77	74.36 ± 8.76	73.99 ± 11.03	0.243
Heart Rate (beats per minute)	71.36 ± 13.82	68.79 ± 11.74	71.68 ± 14.06	0.321
Comorbidities				
Hypertension	67 (52.8%)	9 (64.3%)	58 (51.3%)	0.407
Hyperlipidaemia	56 (44.1%)	7 (50.0%)	49 (43.4%)	0.777
Diabetes Mellitus	16 (12.6%)	1 (7.1%)	15 (13.3%)	0.514
Ischemic Heart Disease	10 (7.9%)	4 (28.6%)	6 (5.3%)	**0.014**
Atrial Fibrillation	10 (7.9%)	3 (21.4%)	7 (6.2%)	0.081
Peripheral Vascular disease	1 (0.8%)	0 (0.0%)	1 (0.9%)	>0.999
Stroke or Transient Ischemic Attack	11 (8.7%)	3 (21.4%)	8 (7.1%)	0.104
Chronic Kidney Disease	6 (4.7%)	4 (3.1%)	3 (2.7%)	0.400
Medication Use				
Antiplatelet	22 (17.3%)	5 (35.7%)	17 (15.0%)	0.067
Oral Anticoagulation	8 (6.3%)	2 (14.3%)	6 (5.3%)	0.215
Beta Blocker	28 (22.0%)	4 (28.6%)	24 (21.2%)	0.508
ACE-I/ARB/ARNI	19 (15.0%)	4 (28.6%)	15 (13.3%)	0.224
MRAs	3 (2.4%)	2 (14.3%)	1 (0.9%)	**0.032**
CCBs	14 (11.0%)	4 (28.6%)	10 (8.8%)	0.049
Diuretics	15 (11.8%)	5 (35.7%)	10 (7.9%)	**0.012**

Abbreviations: ACE-I, angiotensin converting enzyme inhibitor; ARB, aldosterone receptor blocker; ARNI, Aldosterone receptor blocker with neprilysin inhibitor; MRA, mineralocorticoid receptor blocker; CCB, calcium channel blocker.

**Table 2:** Baseline demographics of study population stratified by composite adverse outcome.

Variables	Overall *(n* = 127)	Composite Adverse Outcome (*n* = 54)	Free from Event (*n* = 73)	*p*-Value
Baseline Demographics				
Age (years)	58.46 ± 12.07	58.24 ± 13.33	58.63 ± 11.14	0.056
Male, n (%)	93 (73.2%)	39 (72.2%)	54 (74.0%)	0.842
Height	164.67 ± 8.23	165.17 ± 9.12	164.30 ± 7.50	0.410
Weight	64.83 ± 12.37	64.95 ± 13.60	64.74 ± 11.47	0.123
Smoking	29 (22.8%)	10 (18.5%)	19 (26.0%)	0.394
Systolic Blood Pressure (mmHg)	133.15 ± 18.30	132.33 ± 18.69	133.75 ± 18.12	**0.006**
Diastolic Blood Pressure (mmHg)	74.03 ± 10.77	75.20 ± 10.03	73.16 ± 11.30	0.247
Heart Rate (beats per minute)	71.36 ± 13.82	72.83 ± 2.18	70.27 ± 11.90	**0.042**
Comorbidities				
Hypertension	67 (52.8%)	28 (51.9%)	39 (58.2%)	0.861
Hyperlipidaemia	56 (44.1%)	28 (51.9%)	28 (50.0%)	0.150
Diabetes Mellitus	16 (12.6%)	5 (9.3%)	11 (15.1%)	0.422
Ischemic Heart Disease	10 (7.9%)	6 (11.1%)	4 (5.5%)	0.322
Atrial Fibrillation	10 (7.9%)	7 (13.0%)	3 (4.1%)	0.096
Peripheral Vascular disease	1 (0.8%)	1 (0.8%)	0 (0.0%)	0.425
Stroke or Transient Ischemic Attack	11 (8.7%)	8 (6.3%)	3 (4.1%)	0.053
Chronic Kidney Disease	6 (4.7%)	4 (3.1%)	2 (2.7%)	0.400
Medication Use				
Antiplatelet	22 (17.3%)	12 (27.8%)	7 (9.6%)	**0.009**
Oral Anticoagulation	8 (6.3%)	4 (7.4%)	4 (5.5%)	0.722
Beta Blocker	28 (22.0%)	14 (25.9%)	14 (19.2%)	0.393
ACE-I/ARB/ARNI	19 (15.0%)	7 (13.0%)	12 (16.4%)	0.625
MRAs	3 (2.4%)	2 (3.7%)	1 (1.4%)	0.574
CCBs	14 (11.0%)	7 (13.0%)	7 (9.6%)	0.578
Diuretics	15 (11.8%)	10 (18.5%)	5 (6.8%)	0.054

Abbreviations: ACE-I, angiotensin converting enzyme inhibitor; ARB, aldosterone receptor blocker; ARNI, Aldosterone receptor blocker with neprilysin inhibitor; MRA, mineralocorticoid receptor blocker; CCB, calcium channel blocker.

**Table 3:** Echocardiographic parameters and global left ventricular contractility index stratified by composite outcomes.

Variables	Overall (*n* = 127)	Death (*n* = 14)	*p*-Value	Composite Adverse Outcome (*n* = 54)	*p*-Value
LVEF (%)	64.54 ± 3.10	64.36 ± 4.13	0.813	64.35 ± 3.30	0.552
LVEDD (mm)	54.79 ± 5.85	54.65 ± 5.98	0.470	53.51 ± 5.42	**0.004**
LVESD (mm)	33.14 ± 4.81	33.64 ± 4.22	0.681	34.57 ± 5.02	**0.004**
LVEDV (mm^3^)	148.44 ± 36.63	154.36 ± 29.97	0.524	159.46 ± 38.97	**0.003**
LVESV (mm^3^)	46.06 ± 16.19	47.36 ± 13.72	0.753	51.00 ± 17.48	**0.004**
LVMI (g/m^2^)	121.87 ± 30.79	148.57 ± 35.94	**<0.001**	131.85 ± 34.36	**0.001**
Cardiac Output (L/min)	4.18 ± 1.08	4.11 ± 1.10	0.806	4.13 ± 1.25	0.648
Cardiac Index (L/min/m^2^)	2.44 ± 0.60	2.51 ± 0.55	0.660	2.41 ± 0.67	0.595
LV S’	9.24 ± 2.43	8.36 ± 3.25	0.150	9.12 ± 2.43	0.255
PASP (mmHg)	35.09 ± 9.82	40.69 ± 8.73	0.543	35.50 ± 9.73	0.701
dσ*/dt_max_ (s^−1^)	2.71 ± 0.84	2.19 ± 0.65	**0.013**	2.53 ± 0.84	**0.034**

**Table 4:** Multivariable Cox Regression Analysis of composite outcome.

Factor	HR	*p*-Value
Age	0.993 (0.969–1.017)	0.544
Sex	1.210 (0.646–2.263)	0.354
IHD	1.347 (0.541–3.350)	0.412
PASP	1.001 (0.974–1.038)	0.739
LVESD	1.046 (0.974–1.038)	0.156
Poor LV global contractility	2.056 (1.103–3.835)	0.023

## Data Availability

Data are contained within the article.
